# Rapid sequence induction: where did the consensus go?

**DOI:** 10.1186/s13049-021-00883-5

**Published:** 2021-05-13

**Authors:** Pascale Avery, Sarah Morton, James Raitt, Hans Morten Lossius, David Lockey

**Affiliations:** 1 Southmead Hospital, North Bristol NHS Trust, Bristol, BS10 5NB UK; 2 Essex & Herts Air Ambulance, Flight House, Earls Colne, Colchester, Essex CO6 2NS UK; 3 Thames Valley Air Ambulance Stokenchurch House, Oxford Rd, Stokenchurch, High Wycombe, HP14 3SX UK; 4 Norwegian Air Ambulance Foundation, Holterveien 24, 1441 Drøbak, Norway; 5 Blizard Institute, Queen Mary University, Whitechapel, London, E1 2AT UK

**Keywords:** Rapid sequence induction, Emergency anaesthesia, Standard operating procedures, Apnoeic oxygenation, Video laryngoscopy, Governance

## Abstract

**Background:**

Rapid Sequence Induction (RSI) was introduced to minimise the risk of aspiration of gastric contents during emergency tracheal intubation. It consisted of induction with the use of thiopentone and suxamethonium with the application of cricoid pressure.

This narrative review describes how traditional RSI has been modified in the UK and elsewhere, aiming to deliver safe and effective emergency anaesthesia outside the operating room environment. Most of the key aspects of traditional RSI – training, technique, drugs and equipment have been challenged and often significantly changed since the procedure was first described. Alterations have been made to improve the safety and quality of the intervention while retaining the principles of rapidly securing a definitive airway and avoiding gastric aspiration. RSI is no longer achieved by an anaesthetist alone and can be delivered safely in a variety of settings, including in the pre-hospital environment.

**Conclusion:**

The conduct of RSI in current emergency practice is far removed from the original descriptions of the procedure. Despite this, the principles – rapid delivery of a definitive airway and avoiding aspiration, are still highly relevant and the indications for RSI remain relatively unchanged.

## The background to RSI

Rapid induction of anaesthesia and tracheal intubation is used in the management of critically unwell patients to address the long-recognised risk of aspiration of gastric contents and unnecessary morbidity and mortality [1, 2]. Rapid Sequence Induction (RSI) of anaesthesia was described in 1970 by Stept and Safar. It followed the work by Sellick on the use of cricoid pressure to prevent reflux of gastric contents during induction [1, 3]. The traditional method describes: denitrogenation of the lungs with 100% oxygen for at least 2 min, induction with a pre-determined dose of thiopentone, application of cricoid pressure, administration of a pre-determined dose of suxamethonium, a period of apnoea with no positive pressure ventilation, tracheal intubation with a cuffed tracheal tube, and the release of cricoid pressure when tube placement is successfully confirmed. This led to a remarkably consistent approach to emergency anaesthesia for many years and this technique is still widely practiced in many countries. However, in recent years this consensus has rapidly declined and the emergency anaesthesia literature has revealed considerable variation and controversy in how the intervention is delivered [4–7]. Almost every element of the original technique has been challenged or adapted. This may be because little good evidence has been published since the original description to indicate that traditional RSI effectively reduces aspiration or improves patient outcomes [8]. Alternatively, it may be that emergency anaesthesia has simply evolved with developments in anaesthesia and emergency medicine in terms of technique, training and new drugs and equipment. An ambitious recent systematic review of the limited available high quality evidence attempted to establish which factors improved or failed to improve the success and safety of RSI [9]. It identified key factors as oxygenation strategies, patient position, choice of drugs, checklists and use of videolaryngoscopy. In this narrative review, we will aim to describe how the traditional RSI has evolved and how these and other factors have led to the extensive modification of the standard RSI technique in the UK and elsewhere. We have not specifically addressed RSI in children but many of the same issues apply.

## Methodology

PubMed, Web of Science, Cochrane, OVID, and Embase were searched electronically for relevant articles using Medical Subject Heading (MeSH) terms relevant to each sub-section of the article. For example, “Rapid Sequence Induction OR RSI” AND “checklists OR SOPS OR standard operating procedures OR guidelines”. Titles and abstracts were screened for papers of relevance by two authors (PA and SM). Reference lists of included articles were screened for relevant titles. Articles were then reviewed by two authors (PA and SM) to ensure sufficient quality before being included in the review; if there was any disagreement a third author (DL) was included in the decision.

### The indications for RSI

Although the technique of RSI has evolved, the indications for RSI have remained essentially unchanged for many years. The aim of RSI is to deliver anaesthesia with muscle relaxation to achieve rapid intubation with the prevention of aspiration of gastric contents [10], and most of the many indications for an RSI outside the operating theatre have been categorised using an ABCDE approach (Table 1), with the “A” and “B” indications often recognised as the more common indications [11].

**Table 1:** Indications for RSI

- Airway – loss of airway patency	
- Breathing – inadequate ventilation, respiratory failure or hypoxia	
- Circulation – improve oxygen delivery in hypovolaemia or allow haemorrhage control procedures	
- Disability – neuroprotection particularly in traumatic brain injury, reduced Glasgow Coma Score, status epilepticus, post cardiac arrest protection	
- Everything else – e.g. emergency surgery, humanitarian indications, temperature control (e.g. serotonin syndrome), to facilitate safe transfer.	

### RSI outside the operating theatre

RSI was originally described in an operating theatre environment, but it is no longer only delivered there. Within hospitals, there remains variation between speciality operating theatres, with obstetric theatres still reporting the highest use of a “traditional RSI” [6] among the relatively small numbers of patients receiving general anaesthesia for delivery. The concept of ‘critical care without walls’ – where key critical care interventions are delivered wherever required – has resulted in RSI frequently being performed outside the operating theatre in the emergency department (ED), intensive care unit (ICU) and in pre-hospital settings [12, 13]. The frequently acknowledged challenges with anaesthesia outside the operating theatre include unfamiliar environments, teams, and equipment [14]. The NAP4 study (Major complications of airway management in the United Kingdom) reported that complications outside the operating theatre are common and that one in four major airway adverse events in a hospital are likely to occur in ICU or the ED [15]. Although the case mix of emergency patients anaesthetised outside the operating theatre is always likely to result in higher risk and an increased risk of complications, the number of adverse events may be reduced by improving the standard of airway management. Recent UK guidelines on pre-hospital anaesthesia emphasise that even when anaesthesia is delivered in the pre-hospital phase of care the standards of practice should be similar to those in the emergency department [14].

### Who delivers RSI?

Traditional RSI was delivered by anaesthetists. Emergency anaesthesia is no longer the exclusive reserve of the anaesthetist although in European countries they still deliver the vast majority of procedures. A minority of emergency physicians in the UK have undertaken training in anaesthesia and are able to deliver emergency anaesthesia in the emergency department or as part of a pre-hospital doctor-paramedic team. Emergency anaesthesia conducted by emergency physicians is much more common in some other systems. The Australian and New Zealand Emergency Department Airway Registry (ANZEDAR) reported 3710 intubations carried out over 2 years in 43 emergency departments. 72% were carried out by emergency physicians and the 84.3% first pass intubation success rate is comparable to that reported in studies where intubation was carried out by anaesthetists. The authors do however comment on the significant (26%) adverse event rate. A similar first pass intubation success was reported in Scotland in 3738 patients, 72% of whom were intubated by emergency physicians. In this series a system had developed where emergency physicians and anaesthetists were both present and collaborated in advanced airway management [16].

The UK Royal College of Anaesthetists stipulates that physicians should perform an Initial Assessment of Competence after a 3 month period prior to being able to give anaesthetics without direct supervision [17]. Pre-Hospital Emergency Anaesthesia (PHEA) is usually delivered by a trained team of physician and paramedic and requires additional training, with physicians expected to have completed a minimum of 6 months anaesthetic experience [18, 19]. Drug-assisted paramedic intubation rarely takes place in the UK, although some critical care paramedics may perform advanced airway techniques in a pre-hospital cardiac arrest situation if supraglottic airway devices fail [20]. One US study suggests that at least 25 previous intubation attempts result in an improved first pass intubation rate, with at least 4 weeks of intense training required to achieve this [21].

The evidence reporting intubation success rates and complications by provider type is often of low quality and with considerable heterogeneity between systems. Two meta-analyses of the available evidence demonstrated significantly higher failed intubation rates and adverse events for non-physicians when compared with physicians [22, 23]. This is likely to reflect experience and often low numbers of intubations per provider in emergency pre-hospital practice. Most pre-hospital providers are unlikely to achieve and maintain high success rates without in-hospital training and ongoing practice. Most existing evidence confirms better results with increased experience and numbers of regular intubations [24, 25]. RSI will likely continue to be delivered by an increased variety of providers and the quality of care and intubation success will be dependent on competency influenced by experience, training and relevant continuing professional development. One consistent feature of RSI delivery in high performance systems is that it is delivered by a team rather than an individual provider.

### Checklists and standard operating procedures in RSI

Until relatively recently standard operating procedures and checklists were rarely used in the conduct of RSI in emergency practice. Both are now routinely used in pre-hospital practice and emergency departments in many areas. In a 2017 survey of UK pre-hospital critical care teams that provided RSI, 80% had an RSI SOP and 83% used a checklist [26]. These tools promote a shared mental model of the procedure by the attending team, promote planning for any problems encountered and prevent equipment and preparation errors.

Standard operating procedures (SOP) and checklists have been shown to reduce error and improve patient safety within healthcare systems. One of the best examples of a successful checklist is the World Health Organization (WHO) Surgical Safety Checklist developed by the WHO as part of its Global Patient Safety Challenge. Implementation of the checklist was associated with rapid reductions in mortality and complications among patients who were undergoing surgery [27, 28]. Reductions in complication rates have been reported after the introduction of RSI checklists applied to emergency intubation in the emergency department [29], and in the intensive care unit [30]. However, it is important to understand the limitations of checklist use. They may effectively reduce some complications, for example, equipment and preparation errors but are not a substitute for technical skill and experience. They also do not necessarily modify the presence of difficult patient factors. This may account for some recent studies which report a lack of effect in some clinical endpoints after the implementation of checklists in emergency intubation practice [30–32]. A recent study in pre-hospital practice among experienced Scandinavian providers reported benefits and disadvantages in checklist use as well as highlighting considerable variation in checklist practice [33]. Checklists are likely to remain a key component of emergency intubation, but they are less likely to be effective when used in isolation rather than as a part of a well-rehearsed process conducted by an experienced team [34]. In addition, all checklists are not equal - they need to be carefully designed to be effective. This includes careful consideration of length, content and design to maximise benefit and uptake by providers [35, 36].

Airway guidelines are now widely available and benchmark practice in most countries. Examples are the Difficult Airway Society (DAS) guidelines for in-hospital intubation which include critically ill, obstetric, paediatric and intensive care patients [37, 38]. In the pre-hospital environment guidelines promote simple reproducible techniques combined with checklists and a “challenge-response” approach to ensure steps are not missed [18]. The development of guidelines and checklists delivered as part of a ‘quality bundle’ to cover most aspects of emergency airway management in most clinical areas has been a key factor in the evolution of the conduct of RSI.

### Drugs

The traditional induction agents for Rapid Sequence Induction (RSI) of anaesthesia were thiopentone and suxamethonium, with doses calculated prior to commencing induction [8]. This technique is still used in emergency obstetric anaesthesia [5] although the use of thiopentone without opiates during RSI has been associated with a high risk of accidental awareness [39].

In other emergency practice the use of thiopentone and suxamethonium has become much less common and in some systems disappeared altogether.

Although departure from this standard practice has been rapid it has not been replaced by consistent practice. One relatively common feature of current practice is the addition of an opiate agent to the induction agent and muscle relaxant. Opiates are frequently used as co-induction agents in both hospital and pre-hospital settings, allowing the use of lower doses of hypnotics to promote haemodynamic stability and minimise increases in peri-intubation intracranial pressure. A recent national survey of anaesthetists reported that 80% used an opioid at induction [5, 38].

In the operating theatre setting propofol is now a preferred agent for induction in RSI. Outside the operating theatre the drug combination of fentanyl, ketamine and rocuronium for emergency anaesthesia, particularly in a pre-hospital setting, has become widespread UK practice [40]. The historical concerns regarding ketamine and raised intracranial pressure have now been widely rejected [41, 42] and there are guidelines which favour the use of ketamine in critically ill patients due to its haemodynamic stability [38]. While ketamine use has increased, the use of etomidate has sharply decreased in many countries in the same period. Etomidate was considered an induction agent with minimal haemodynamic effects but has fallen out of favour in hospital and critical care because of its adrenocortical suppression side effects. There is little evidence for harmful adrenocortical suppression after a single induction dose of etomidate but it seems unlikely that use of this agent will increase back to previous levels [43].

The importance of “adequate neuromuscular blockade” is a key point stressed in the DAS airway guidelines to avoid difficult intubating conditions [37, 44]. The reduction of the use of the short acting depolarizing muscle relaxant suxamethonium which was associated with excellent intubating conditions has been dramatic. This was driven by the introduction of rocuronium into anaesthetic practice which, when administered at high doses, has a much more rapid onset of action than older non-depolarizing agents. Studies comparing the two agents have failed to show significant differences in intubation success or complications [44], although a Cochrane review in 2015 still narrowly favoured the use of suxamethonium for RSI [45]. In the same year a survey of UK pre-hospital practice reported 41% of services using rocuronium for RSI and this has likely increased significantly in subsequent years [46]. The development of sugammadex, an agent which can rapidly reverse the effects of rocuronium [47] has been suggested as a reason why RSI is now frequently conducted with rocuronium. In fact, rocuronium was being used for RSI before the introduction of sugammadex and a large proportion of critically ill patients are not in the patient population where ‘waking’ after failed intubation is an option [4]. Sugammadex is rarely carried by pre-hospital services [46]. The confidence to use a long acting muscle relaxant may be increased by the very high success rates of airway rescue techniques reported after failed intubation in recent large studies, even in the resource-poor pre-hospital environment [46, 48].

Drug use for RSI has not only changed significantly since the original descriptions of RSI but internationally reported practice demonstrates major variations. A prospective study of 3710 medical and trauma intubations in 43 emergency departments in Australia and New Zealand in 2017 mostly by emergency physicians reported that propofol, thiopentone or ketamine were used with similar frequency for induction, and suxamethonium was still the most commonly used muscle relaxant [49]. In German pre-hospital practice when a total of 9720 RSIs were examined over a 10 year period dramatic reductions in etomidate were reported with a rise in propofol and ketamine use [50]. Fentanyl was the most commonly administered opiate but sufentanil was also increasingly used. These studies demonstrate that even within countries the use of drugs for RSI are very varied and there is little consensus on which combination of agents is ideal. An alternative view might be that expert practitioners are increasingly tailoring their anaesthetic techniques to specific patient groups.

### Pre-induction sedation

Patients requiring emergency anaesthesia are sometimes agitated or noncompliant due to trauma or critical illness. In order to achieve safe and effective conditions for monitoring, pre-oxygenation and induction of anaesthesia it may be necessary to sedate the patient with a small dose of the intended induction drug or another sedative agent. This is essentially procedural sedation to achieve suitable conditions for induction of anaesthesia. This technique is in conflict with traditional RSI where no agents are administered before induction but was described in UK pre-hospital anaesthesia guidelines in 2009 and again in 2017 [18]. A safety feature of the technique is that sedation is only administered when preparation for RSI is completed.

### Cricoid pressure

Cricoid pressure was introduced into clinical practice as a key element of RSI in 1961, based mainly on a small case series on cadavers [1]. It is used to compress the oesophagus and prevent regurgitation of gastric contents until the airway is secured with a tracheal tube. Many anaesthetists also use it to manipulate the upper airway to improve laryngeal view. A pressure of 20-40 N is cited as the amount of pressure that should be applied [10]. There is a growing scepticism about the efficacy of cricoid pressure during emergency anaesthesia. A Cochrane review published in 2015 noted the lack of high quality evidence on the subject but acknowledged that what evidence there was did not strongly support the intervention [51]. Subsequently a multicentre randomized, double-blind, study conducted in the US was published in 2019 and demonstrated that in 3472 patients who had emergency RSI the use of cricoid pressure did not reduce the incidence of aspiration. It also suggested that intubation was more difficult in the cricoid pressure group [52]. There is a school of thought that if cricoid pressure is used it may inhibit air entry into the stomach. Therefore ventilation during the apnoeic period following drug administration may be less likely to provoke aspiration. The UK is one of only a few countries with a high use of cricoid pressure particularly compared to other European countries [53]. UK DAS guidelines recommend the early removal of cricoid pressure if there is a difficult laryngoscopy [37]. Other national and international guidelines including those by the European Resuscitation Council, Scandinavian Clinical Practice Guidelines, and German Airway Management Guidelines do not support its use [54–56].

The use of cricoid pressure as an essential component of RSI is decreasing. When it is used it is widely recommended that it should be rapidly released in the event of a poor view at laryngoscopy.

### Peri-induction oxygenation

There is recognition that the balance of risk between aspiration and hypoxia was not addressed by the traditional RSI method. This is particularly important for critically ill patients who are at high risk of becoming hypoxic during the apnoeic period of induction [57]. This has led to the development of simple techniques to maintain oxygenation.

Nasal apnoeic oxygenation can be delivered by high flow oxygen (10-15 L/min) through nasal cannulae and has been shown to significantly increase the time to desaturation during the apnoeic period of induction [58, 59]. The development of transnasal humidified rapid-insufflation ventilatory exchange (THRIVE) has also been used to maintain oxygenation although mostly in the operating theatre environment [60, 61]. In non-operating theatre environments, it has been clearly demonstrated that apnoeic oxygenation is straightforward to deliver. Where the airway is patent it would be fully expected to increase the time to desaturation and this has been confirmed in the pre-hospital environment [62]. Unfortunately, clear benefit has not been demonstrated in more recent studies [63, 64]. On the basis that apnoeic oxygenation is likely to delay the onset of hypoxaemia in some patient groups and is easy to deliver, it is likely to continue to be used in emergency practice. A different approach to the prevention of hypoxia in the apnoeic period is to avoid apnoea during induction altogether. A large multicentre trial showed that gentle bag valve mask ventilation between induction and laryngoscopy reduced the incidence of hypoxia without adversely affecting aspiration rates [65]. This practice may already have been commonplace. In 2016, 17% of UK anaesthetists surveyed performed gentle mask ventilation during the apnoeic period during emergency RSI [5].

Another simple technique to improve oxygenation during emergency anaesthesia is to optimise patient positioning. In addition to improving oxygenation a head up position has been recommended to prevent aspiration and improve intubation success [38]. However, a recent study randomised patients undergoing emergency RSI to a ‘25^o^ ramped up’ position and compared them to patients in the standard supine position. The authors were unable to demonstrate any benefits in oxygenation and also reported significantly worse laryngoscopic views and increased intubation difficulty [66]. The benefits or disadvantages of a head up position are not therefore clear. There are other positioning considerations which can also influence intubation difficulty including removal of cervical collars and ensuring, in the pre-hospital environment, 360-degree access to the patient and the use of an ambulance trolley at waist height to optimise laryngoscopy attempts [18]. The ability to tip a trolley is also desirable [10].

Simple strategies to maintain oxygenation and improve the laryngoscopic view have been incorporated into emergency RSI techniques. Although the benefits have not been straightforward to establish, the available evidence suggests that adaptations do not cause increased risk of complications such as aspiration.

### Equipment developments

#### Laryngoscopy

The increasing availability and portability of video laryngoscopes has led to a significant increase in their use in emergency anaesthesia including in emergency departments and pre-hospital care. A Cochrane review published in 2016 examined over 60 studies and concluded that video laryngoscopy can improve laryngoscopic view and reduce failed intubation rates particularly in patients with difficult airways. Only three studies included emergency patients [67]. The published evidence for the value of video laryngoscopy over direct laryngoscopy in emergency anaesthesia is inconclusive and the quality of available evidence is low [67]. Indeed, one study showed no difference between direct and video laryngoscopy in survival to hospital discharge in trauma patients and showed a longer median time to intubation; sub-group analysis of severe head injury patients seemed to be associated with a greater incidence of hypoxia during the intubation attempt [68]. There is however consensus that, in the airway management of critically ill patients videolaryngoscopy should be immediately available [38]. The benefits of availability were confirmed in a recent randomised pre-hospital study which found both direct and indirect laryngoscopy to be equally effective but reported that swapping from one technique to the other was more successful than a second attempt with the same device [69].

Despite the fact that intubation difficulties are more likely to occur outside the operating theatre environment a recent survey reported that video laryngoscopes are most commonly available in operating rooms and less so in the areas where emergency anaesthesia is performed outside the operating room [70]. The potential risks of transmission of infection during emergency intubation have been brought into sharp focus during the COVID-19 pandemic. UK guidelines recommend videolaryngoscopy as the first line option for emergency intubation in high risk patients [71].

#### Intubating bougie

Since the description of RSI the use of an intubating bougie has become standard UK practice to optimise the first attempt of emergency tracheal intubation during both direct and video laryngoscopy [19, 72, 73]. The simple device is used variably in other countries, but some recent high-quality studies have demonstrated superiority over other intubation techniques [74].

#### Monitoring

Advances in the standard of monitoring equipment have transformed the conduct of emergency anaesthesia. There are well developed minimum monitoring national standards in place for elective and emergency anaesthesia and almost identical standards are recommended for pre-hospital anaesthesia [18]. The use of capnography to detect oesophageal intubation is a key improvement in the conduct of RSI and has been identified as a vital tool in the prevention of life threatening complications in emergency intubation [15, 75]. In patients requiring neuroprotection, invasive blood pressure monitoring may be indicated to prevent both hypo and hypertension [76].

### Management of failed intubation after RSI

The developments in equipment, monitoring and techniques have made failed intubation after RSI less common. When intubation does fail rescue techniques have developed to make poor outcomes less likely. The development of supraglottic airways have made a proportion of ‘can’t intubate, can’t ventilate’ situations much easier to manage and the use of familiar failed intubation guidelines have reduced hypoxic time and the speed at which rescue techniques, including rescue surgical airway, are performed [37]. Recent studies have also suggested a fall in the rates of rescue surgical airway both in emergency departments [77] and in pre-hospital practice [78].

### Governance of RSI

In most well-resourced hospitals and emergency medical systems the conduct of RSI is subject to multiple governance processes which were not present when RSI was introduced. Training, supervision, guidelines, and incident reporting are just some of the governance elements which aim to deliver safer emergency anaesthesia. Registries and databases provide information to guide quality improvement and major multicentre national audit projects have identified key areas for improvement of the RSI process, particularly around airway complications and awareness [15, 39]. More recently this activity has extended to more the difficult areas of emergency anaesthesia in the emergency department and in pre-hospital care [14] and individual services have adopted quality improvement recommendations such as key performance indicators for pre-hospital anaesthesia [75].

## Conclusions

In many respects the conduct of RSI in current emergency practice is far removed from the original descriptions of the procedure. Despite this, the principles – rapid delivery of a definitive airway and avoiding aspiration, are still highly relevant and the indications for RSI remain relatively unchanged. Changes to the procedure have tackled several considerations less well addressed by the original technique including reducing the frequency and severity of hypoxaemia, reducing the frequency of failed intubation and making detection and management of complications more effective. The remarkable consensus in RSI practice that persisted for many years has lessened in recent years. Despite this, standardisation is often in place in many systems. It is difficult to know whether variations in practice are necessary to deliver tailored care to different patient groups or whether increased consistency has the potential to improve overall patient safety. In in-hospital practice it has been suggested that avoidance of adverse events and successful intubation of critically ill patients on the first attempt can be positively influenced by operator related factors including training and experience, equipment selection and drug choices [79]. Limiting choice and delivering a very standard RSI might be more appropriate when RSI is delivered by less experienced operators in more austere environments [14]. One consistency in high performing systems is the recognition that the delivery of high-quality RSI is not a solo activity and requires an effective team approach to apply the appropriate techniques. In addition, attention to all factors in RSI delivery is important. Systems reporting improvement rarely introduce or change only one component of the procedure. Usually a ‘bundle’ of improvements has been required to address all aspects of the procedure and deliver improved quality [34, 80].

The safety of RSI is as important now as it was when first described. It is carried out on our sickest and most unstable patients in all emergency treatment areas. Choices of drugs and techniques have rapidly increased and many of these changes have the potential to improve safety. Providers of RSI now have a wide range of tools and techniques available to enhance the basic procedure. These can be incorporated into a structured plan to deliver safe emergency anaesthesia to their particular patient casemix.

## Data Availability

Data sharing not applicable to this article as no datasets were generated or analysed during the current study.

## Abbreviations

RSI: Rapid Sequence Induction
DAS: Difficult Airway Society
GCS: Glasgow Coma Score
WHO: World Health Organisation
SOP: Standard Operating Procedures
PHEA: Pre-Hospital Emergency Anaesthesia ED Emergency Department
ICU: Intensive Care Unit
NAP: National Audit Project
